# Oxidative stress in lung cancer patients is associated with altered serum markers of lipid metabolism

**DOI:** 10.1371/journal.pone.0215246

**Published:** 2019-04-11

**Authors:** Katarzyna Zabłocka-Słowińska, Sylwia Płaczkowska, Katarzyna Skórska, Anna Prescha, Konrad Pawełczyk, Irena Porębska, Monika Kosacka, Halina Grajeta

**Affiliations:** 1 Department of Food Science and Dietetics, Wroclaw Medical University, Wroclaw, Poland; 2 Diagnostics Laboratory for Teaching and Research, Wroclaw Medical University, Wroclaw, Poland; 3 Department and Clinic of Thoracic Surgery, Wroclaw Medical University, Wroclaw, Poland; 4 Department and Clinic of Pulmonology and Lung Cancers, Wroclaw Medical University, Wroclaw, Poland; University College London, UNITED KINGDOM

## Abstract

In lung cancer (LC), alterations in redox balance are extensively observed and are a consequence of disease as well as co-occurrent with smoking. We previously demonstrated that metabolic disturbances such as trace element status and carbohydrate metabolism alterations are linked with redox status. The aim of this study was to evaluate relationships between the serum parameters of lipid metabolism and redox balance in LC patients. Serum parameters of lipid metabolism, i.e. total cholesterol (T-C), HDL cholesterol (HDL-C), LDL cholesterol (LDL-C), triglycerides (TG), T-C:HDL-C ratio, non-HDL-C, apolipoprotein A1 (Apo-A1), apolipoprotein B (Apo-B) and Apo-B:Apo-A1 ratio, as well as systemic redox status, i.e. total antioxidant status (TAS), total oxidant status (TOS), oxidative stress index (OSI), vitamin E (VE), vitamin C (VC), malonyldialdehyde (MDA), conjugated dienes (CD), and 4-hydroxynonenal (4-HNE) were determined in 92 LC patients and 82 control subjects (CS). LC women had significantly lower T-C and LDL-C, and higher TG, while HDL-C, Apo-A1 and Apo-B were significantly decreased in LC patients regardless of sex, when compared to CS. LC men had alterations in the systemic total redox balance such as lower TAS and higher OSI than CS men. LC women had lower VC, but VE was decreased in LC patients, regardless of sex. We observed higher lipid peroxidation in LC patients expressed via higher 4-HNE and CD. Systemic redox disturbances were associated with serum lipid alterations: TOS and OSI were positively correlated with T-C:HDL-C ratio and Apo-B:Apo-A1 ratio and negatively with HDL-C. The parameters of lipid peroxidation CD and MDA were significantly associated with variables reflecting lipid disturbances. The observed correlations were strengthened by general overweight/obesity, abdominal obesity, hypertriglyceridemia and non-smoking status. In conclusion, parameters related to lipid alterations are associated with oxidative stress in LC patients. The largest contribution from lipid parameters was revealed for T-C:HDL-C ratio, HDL-C and Apo-B:Apo-A1 ratio, while the largest contribution from redox status was revealed for OSI and VE. Overweight, obesity, hypertriglyceridemia and non-smoking status intensified these relationships.

## Introduction

Disturbances in anti-/prooxidant balance resulting in oxidative stress have been recognized and intensively studied over the last several decades as one of the main factors contributing to chronic inflammation. Systemic, long-term, persistant inflammation predisposes people to major, chronic, non-infectious diseases including cardiovascular, diabetic, neurological, cancer and pulmonary diseases [1]. In lung cancer, the most common malignancy worldwide, chronic lung inflammation is proposed as one of the main endogenic risk factors, regardless of smoking status. Nevertheless, cigarette smoking still remains the main exogenic determinant promoting the generation of reactive oxygen species and thus inducing inflammatory-related airway injuries [2]. Several studies have also indicated that lung cancerogenesis is directly linked with redox imbalance, both in organs and systemic [3,4]. Although the link between cigarette smoking and redox imbalance in lung cancer has been extensively studied and is widely known, factors other than tobacco smoke may contribute to systemic oxidative stress in lung cancer patients, since prevalence of the disease is systematically increasing also among non-smokers [5]. In our previous studies, we proved that alterations in redox balance in lung cancer may also be influenced by endogenous metabolic disturbances, e.g. alterations in glucose metabolism [3], as well as trace element status disorders [6]. Alterations of lipid metabolism in lung cancer patients have been demonstrated in several cohort and experimental studies and have even been considered as potential risk factors [7–10]. Patients with lung cancer often demonstrate lower serum total cholesterol (T-C), as well as HDL cholesterol (HDL-C) and LDL cholesterol (LDL-C), while levels of triglycerides (TG) have been recognized as increased in this condition [7,8]. As confirmed by other authors in studies performed in different populations, both ill and healthy, alterations in serum markers of lipid metabolism may contribute to oxidative stress. In fact, some components of lipid metabolism, e.g. HDL-C, may present direct antioxidant activity [11], while the level of modification of others, e.g. LDL-C and TG, may indirectly protect from oxidative stress and inflammation [11–13]

Based on the above-presented information, that alterations in lipid metabolism may influence systemic redox status, and the lack of such studies in different cancer populations, including lung cancer, we aimed to evaluate relationships between serum markers of lipid metabolism and parameters related to redox balance in lung cancer patients.

## Material and methods

### Lung cancer patients and control subjects

One hundred and seventy-four participants were enrolled in the study. Newly diagnosed lung cancer patients, (n = 92) were recruited from the Lower Silesian Centre of Lung Diseases after confirmed diagnosis and before any oncological treatment. The clinical stages of disease were evaluated based on chest computed tomography, PET-CT and ultrasonography of the abdominal cavity. CT/MRI of the central nervous system and bone scintigraphy were performed if necessary. Bronchofiberoscopy was carried out routinely. In the case of enlarged lymph nodes of the mediastinum, endobronchial ultrasound transbronchial needle aspiration (EBUS-TBNA) was performed. Negative EBUS results were verified by mediastinoscopy.

The control subjects (n = 82) consisted of healthy people recruited from the Center of Occupational Medicine and Wroclaw University of the Third Age. Exclusion criteria for the control group were as follows: presence of any chronic disease, especially cancers, pro-inflammatory diseases, neurological disorders and mental health issues, and the use of any medical agents or dietary supplements. Patient and control subjects were characterized in terms of cigarette smoking status, alcohol consumption—defined as at least one portion (20 g of ethanol) per month, exposure to chemical agents (mainly in the workplace), place of residence (village, small city <200,000 inhabitants, big city ≥ 200,000 inhabitants), education level (years of education), anthropometric parameters and dietary intakes. Additionally for the group of lung cancer patients, data on co-morbidities and chronically used medicines were obtained from patient medical cards prepared by their doctors.

Detailed data on lung cancer patients and control subjects are summarized in Table 1.

**Table 1:** Baseline characteristics of sex-related groups of lung cancer patients (n = 92) and control subjects (n = 82) [median (Q1 –Q3)].

Variables	Women				Men			
	N	Lung cancer (n = 43)	n	Control (n = 42)	N	Lung cancer (n = 49)	n	Control (n = 40)
**Age [years]**	43	63.0 (58.0–67.0)	42	63.0 (56.0–66.0)	49	62.0 (59.0–65.0)***	40	49.5 (45.5–60.0)***
**Smoking status; yes/no/previous**	43	23.3/39.5/37.2	42	11.9/54.8/33.3	49	32.7/30.6/36.7	40	42.5/40.0/17.5
**Alcohol consumption: Y/N [%]**	43	44.2/55.8	42	64.3/35.7	49	65.3/34.7*	40	85.0/15.0
**Portions of alcohol consumed per month:<1/1-5/6-10/>10 [%]**	43	55.8/41.9/2.3/0.0	42	35.7/54.8/7.1/2.4	49	34.7/34.7/22.5/8.2	40	15.0/52.5/20.0/12.5
**Chemical agent exposure: Y/N [%]**	43	9.3/90.7	42	14.3/85.7	49	44.9/55.1	40	37.5/62.5
	**Clinical data**							
**Histological type; NSCLC/SCLC/no data [%]**	43	74.4/7.0/18.6	-	-	49	87.8/6.1/6.1	-	-
**Clinical stage of disease; I/II/III/IV/no data [%]**^**1**^	43	32.6/20.9/11.6/9.3/25.6	-	-	49	28.6/20.4/14.3/26.5/10.2	-	-
**Comorbidities: asthma/COPD/atherosclerosis/hypertension/kidney diseases/T2DM [%]**^**1**^	43	2.3/16.3/0.0/27.9/7.0/18.6	-	-	49	0.0/16.3/14.3/34.7/4.1/14.3	-	-
**Chronically used drugs: inhaled /antihypertensive/statins/antidiabetic/NSAID [%]**		11.6/16.3/4.6/16.3/4.7		-		6.1/28.6/16.3/14.3/2.0		-
**Education level: ≤11 y/12-16 y/≥17 y[%]**	43	34.9/53.5/11.6**	42	7.1/59.5/33.3	49	63.3/28.6/8.1	40	42.5/35.0/22.5
**Place of residence: village/small city/big city**	43	18.6/53.5/27.9***	42	9.5/7.1/83.3	49	24.5/55.1/20.4**	40	25.0/20.0/55.0
	**Anthropometric parameters**							
**Weight [kg]**	43	73.0 (54.0–82.0)	42	69.6 (60.0–81.0)	49	78.0 (65.0–85.0)*	38	85.0 (78.0–92.0)
**BMI [kg/m**^**2**^**]** ^**W**^	42	28.2 (23.2–32.0)	41	26.8 (23.6–30.0)	49	25.6 (22.8–28.4)	38	27.1 (25.2–28.7)
**BFP [%]** ^**W,M**^	37	33.8 (28.7–39.2)	30	36.2 (32.4–40.4)	46	24.8 (17.9–29.3)	20	24.5 (21.7–28.3)
**WC [cm]**	37	96.5 (82.5–106.5)	38	86.0 (78.0–98.0)	46	94.0 (85.0–104.0)	32	98.2 (93.0–103.0)
**HC [cm]** ^**W**^	36	106.5 (93.5–115.0)	37	102.0 (98.0–108.0)	46	98.0 (90.0–103.0)**	**32**	103.5 (98.0–108.0)
**WHR** ^**M**^	36	0.91 (0.85–0.95)*	37	0.84 (0.79–0.90)	46	0.97 (0.94–0.99)	32	0.94 (0.91–1.01)
	**Nutrient intakes**							
**Energy [kcal/d]**	42	1833.3 (1536.3–2117.9)***	42	1664.0 (1432.3–1977.5)	49	2573.7 (2118.9–3305.2)**	38	2246.3 (1596.9–2665.5)
**Energy intake from: carbohydrate/fat/protein [%]; median**	42	48.8/27.6/13.1	42	55.4/29.8/16.5	49	52.8/32.8/15.7	38	52.3/33.6/16.5
**Total protein [g/d]**	42	66.5 (57.2–79.1)	42	68.0 (55.7–75.0)	49	108.0 (78.4–126.9)	38	94.6 (73.4–109.3)
**Total carbohydrate [g/d]**	42	248.9 (213.7–318.1)	42	224.7 (181.3–272.9)	49	329.8 (272.8–433.5)*	38	260.5 (221.0–343.2)
**Total fat [g/d]**^**M**^	42	64.7 (53.3–74.9)	42	51.3 (45.0–71.2)	49	99.3 (72.1–130.5)*	38	81.3 (57.6–107.7)
**SFAs [g/d]**^**M**^	42	25.6 (19.5–33.4)**	42	19.1 (13.9–28.8)	49	38.6 (30.2–49.4)	38	32.3 (22.3–43.5)
**MUFAs [g/d]** ^**M**^	42	23.6 (19.9–28.8)	42	21.2 (17.5–27.9)	49	37.7 (27.4–53.5)*	38	32.0 (20.1–41.9)
**PUFAs [g/d]**	42	8.9 (6.5–10.9)	42	8.7 (7.1–10.7)	49	13.0 (10.1–15.9)	38	11.1 (7.1–14.6)
**LC-PUFAs [mg/d]**	42	106.3 (31.0–341.7)	42	126.5 (40.0–241.5)	49	269.9 (44.5–706.5)	38	77. 0 (62.2–310.9)
**EPA [g/d]**	42	0.029 (0.005–0.120)	42	0.031 (0.004–0.070)	49	0.077 (0.003–0.236)	38	0.022 (0.003–0.113)
**DHA [g/d]**	42	0.079 (0.025–0.181)	42	0.092 (0.034–0.159)	49	0.193 (0.044–0.405)	38	0.067 (0.046–0.214)
**Cholesterol [mg/d]**	42	256.1 (202.0–314.3)	42	273.9 (179.8–345.2)	49	431.7 (303.8–494.6)	38	342.7 (276.4–457.2)
**Vitamin C [mg/d]**	42	90.1 (75.0–116.3)	42	115.5 (83.4–154.4)	49	123.5 (88.6–152.7)	38	94.8 (60.2–176.1)
**Vitamin E [mg/d]**	42	8.0 (7.0–10.8)	42	8.4 (6.6–11.5)	49	11.0 (7.6–13.6)*	38	8.8 (5.7–10.8)
**Retinol [μg/d]**	42	374.6 (287.2–472.2)*	42	321.8 (201.1–427.5)	49	501.7 (344.4–648.9)	38	386.8 (265.8–563.9)
**B-carotene [μg/d]**	42	3393.6 (2412.8–4579.2)*	42	4567.6 (3008.0–7880.8)	49	4008.3 (2945.2–5290.2)	38	3921.6 (2573.5–4971.2)

Y–yes; N–no

^1^ –percentages do not sum up to 100% due to multiple choices

NSCLC–non-small cell lung cancer; SCLC–small cell lung cancer; COPD–chronic obstructive pulmonary disease; T2DM–type 2 diabetes mellitus; NSAID–non-steroidal anti-inflammatory drugs; BFP–body fat percentage; WC–waist circumference; HC–hip circumference; WHR–waist-hip ratio; SFAs–saturated fatty acids; MUFAs–monounsaturated fatty acids; PUFAs–polyunsaturated fatty acids; LC-PUFAs–long chain polyunsaturated fatty acids; EPA–eicosapentaenoic acid; DHA–docosahexaenoic acid; significant differences in biochemical parameters between sex-related groups are bolded

*p<0.05

** p<0.01

***p<0.001 -differences in age, anthropometric parameters, nutrient intakes between lung cancer and control sex-related groups were evaluated with t-Student or U-Mann-Whitney test, depending on data distribution.

^W,M^–women, men -sex-related groups for whom parameters were evaluated with t-Student test (normally distributed) are marked in the first column; Chi2 test was used for intergroup comparisons of smoking status, alcohol consumption, chemical agent exposure and place of residence

### Methods

#### Anthropometric measurements and dietary intake

Anthropometric parameters, as well as energy and nutrient intakes, were used for the nutritional status assessment of participants. Baseline anthropometric parameters were measured: weight, body fat percentage (BFP), waist and hip circumferences. Waist circumference (WC) was measured at the level of the umbilicus, and hip circumference (HC) at the trochanter levels. The percentage of body fat was determined using a Body FAT Monitor (Omron BF 306, Japan). WHR was calculated as the ratio of WC and HC. Body mass index (BMI) was calculated from weight and height. All anthropometric measurements were performed twice and the mean was used for further analysis.

Dietary data were gathered using three 24-h dietary recalls by a trained interviewer. In the case of lung cancer patients this took place on the first day of admission to hospital. To assess information about the portion size of food products, an album of photographs of food products and dishes (National Food and Nutrition Institute, Warsaw, Poland) was used [14]. All dietary recalls were analyzed using Dieta 5.0 (National Food and Nutrition Institute, Warsaw, Poland).

#### Blood samples collection and preparation

After overnight fasting, blood samples were collected from all participants and serum was separated. In the case of lung cancer patients, blood was collected after admission to hospital. All material was stored at -80°C until analysis. The study protocol was approved by the Ethics Commission of Wroclaw Medical University (approval no. 540/2013), and the study was conducted according to the principles expressed in the Declaration of Helsinki. All participants provided written consent for taking part in the research.

#### Parameters measured in serum, related to systemic redox status

**Total antioxidant status (TAS).** Total antioxidant status (TAS) was measured using a commercial TAS kit (Randox Laboratories, UK). The procedure was carried out following the manufacturer’s instruction. The analysis performed in serum is based on incubation of 2,2′-azino-di-(3-ethyl-benzthiazoline sulfonate) (ABTS) with a peroxidase (metmyoglobin) and H_2_O_2_ to produce the radical cation ABTS^+^. This cation has a relatively stable blue-green color. The blood antioxidants cause cationic neutralization and the disappearance of color, which is measured at 600 nm. The assay is calibrated with Trolox and the results are expressed in terms of mmol/L Trolox.

**Total oxidant status (TOS).** Total oxidant status (TOS) was measured as described by Erel [15]. Using this method, the oxidants present in the sample oxidize the ferrous ion-o-dianisidine complex to the ferric ion. The ferric ion produces a colored complex with xylenol orange in an acidic medium. The color intensity is measured at 560 nm and is related to the total amount of oxidant molecules present in the sample. Briefly, the first absorbance is measured after mixing serum (35 **μ**l) with reagent 1 (orange 150 **μ**M, NaCl 140 mM and glycerol 1.35 M in 25 mM H_2_SO_4_ solution, pH 1.75) as a sample blank, and the second after adding reagent 2 (ferrous ion 5 mM and *o*-dianisidine 10 mM in 25 mM H_2_SO_4_ solution) when the reaction reaches a plateau (3 min after mixing). The assay was calibrated with hydrogen peroxide and the results are expressed in terms of **μ**mol H_2_O_2_ equivalent/L of serum.

**Oxidative stress index (OSI).** The TOS:TAS ratio was used as the oxidative stress index (OSI), and calculated as follows [16]:

$$\mathrm{OSI}\left(\mathrm{arbitrary}\;\mathrm{units}\right)=\frac{\mathrm{TOS}\;\left[{\mu \mathrm{mol}\;H}_{2}O_{2}/L\right]}{\mathrm{TAS}\;\left[\mathrm{mmol}\;\mathrm{Trolox}\;\mathrm{equiv}./L\right]}$$

**Malondialdehyde (MDA).** MDA was measured based on reaction with thiobarbituric acid (TBA) and extraction with 1-butanol after heating in 95°C water for 45 minutes. The absorbance of the pink supernatant was measured at 535 nm and results were calculated using the molar coefficient and expressed in μmol of MDA/L of serum.

**4-hydroxynonenal (4-HNE).** 4-HNE was measured using a human 4-hydroxynonenal ELISA kit (Cusabio) according to the manufacturer’s instructions. Briefly, the microtiter plate was pre-coated with an antibody specific to 4-HNE. Standards and 200-fold diluted samples were added to the appropriate wells with horseradish peroxidase (HRP) conjugated 4-HNE. The competitive inhibition reaction was launched between HRP-conjugated 4-HNE and 4-HNE in samples. After adding substrate solution, the color developed in inverse proportion to the amount of 4-HNE in the samples. The results were expressed on the basis of a calibration curve.

**Conjugated dienes (CD).** CD were measured spectrophotometrically, based on the Recknagel et al. [17] method. After extracting lipid from sera with a chloroform-methanol (2:1) mixture and centrifugation, the chloroform layer was separated and evaporated in a nitrogen atmosphere. Then the residues after evaporation were dissolved in cyclohexane and the absorbance of peroxides was measured at 234 nm. Concentrations of CD were measured according to the following formula and expressed as μmol of conjugated dienes/L of serum:

$$\mathrm{CD}\;\left[\mu \mathrm{mol}/L\right]=\frac{{\mathrm{Abs}\;x\;V}_{\mathrm{cyclohexane}}}{0.28\;x\;V_{\mathrm{serum}}}$$

**Vitamin E (VE).** VE concentration was measured with a commercial ELISA kit (Cloud-Clone Corp.). The procedure was carried out following the manufacturer’s instruction. A monoclonal antibody specific to alpha-tocopherol was pre-coated onto a microplate. A competitive inhibition reaction was launched between biotin labeled alpha-tocopherol and unlabeled alpha-tocopherol from standard or samples with a pre-coated antibody specific to alpha-tocopherol. After incubation the unbound conjugate was washed off. Then, avidin conjugated to horseradish peroxidase (HRP) was added to wells and incubated. The amount of bound HRP conjugate is inversely proportional to the concentration of alpha-tocopherol in sample sera. After addition of the substrate solution the intensity of the color developed was measured at 450 nm. Results were expressed on the basis of a calibration curve.

**Vitamin C (VC).** VC concentration was measured using a commercial ELISA kit (Sunred Biological Technology, Shanghai, China). The procedure was carried out following the manufacturer’s instruction. The kit uses a double-antibody sandwich ELISA. Briefly, vitamin C was added to the monoclonal antibody enzyme well, which was pre-coated with human VC monoclonal antibody. After incubation, VC antibodies labeled with biotin were added and combined with streptavidin-HRP to form an immune complex. Then incubation was carried out and the uncombined enzyme was removed. Next, chromogen solutions A and B were added and a color was developed which was directly proportional with serum VC concentration. The intensity of color was measured at 450 nm. The results were expressed on the basis of a calibration curve.

#### Parameters measured in serum related to lipid metabolism

**Total cholesterol (T-C).** Total cholesterol (T-C) was determined by the spectrophotometric method, after enzymatic hydrolysis of cholesterol esters to cholesterol and fatty acids. The procedure was carried out following the manufacturer’s instruction (DiaSys Germany). Cholesterol was then oxidized to cholesterol-3-one and H_2_O_2_, then peroxidase catalyzed the reaction of H_2_O_2_ with 4-aminoantipyrine and phenol, forming a colored product–quinone imine. The intensity of the color measured at 500 nm is proportional to the serum cholesterol concentration.

**HDL cholesterol (HDL-C).** The HDL-C fraction was determined by the immunochemical method using antibodies against human lipoproteins that form complexes with LDL-C, very-low-density lipoprotein (VLDL-C) and chylomicrons. The procedure was carried out following the manufacturer’s instruction (DiaSys Germany). HDL-C was measured by the spectrophotometric method, after enzymatic hydrolysis and oxidation, to cholest-4-en-3-one and H_2_O_2_. Then peroxidases catalyzed the reaction between H_2_O_2_, sodium salt of N-ethyl-N- (2-hydroxy-3-sulfopropyl) -3,5-dimethoxy-4-fluoroaniline and 4-aminoantipyrine, forming a blue complex. Absorbance was measured at 600 and 700 nm. The first measurement was performed after the formation of lipid complexes with antibodies (A1), and the second after HDL-C reactions leading to the formation of a colored complex (A2). Absorbance for HDL-C was calculated with the formula:

$$A_{\mathrm{HDL}‐\mathrm{Ch}}=A2–A1$$

**LDL cholesterol (LDL-C).** LDL-C was calculated using Friedwald equation [18]:

$$\mathrm{LDL}‐C\;\left[\mathrm{mg}/\mathrm{dl}\right]=T‐C-\left(\mathrm{HDL}‐C+\frac{\mathrm{TG}}{5}\right)$$

**Triglycerides (TG).** TG were determined by the spectrophotometric method, following the manufacturer’s instruction (DiaSys Germany), after enzymatic hydrolysis to glycerol and fatty acids. Glycerol then reacted with adenosine triphosphate (ATP) and glycerokinase to form glycerol-3-phosphate and adenosine diphosphate (ADP). Oxidase catalyzed oxidation of glycerol-3-phosphate to dihydroxyacetone phosphate and H_2_O_2_. Peroxidase catalyzed the reaction of H_2_O_2_ with aminoantipyrine and 4-chlorophenol, forming the colored product quinone imine measured at 500 nm. The intensity of the color was proportional to the quantity of glycerol in the sample. The results were corrected for the free glycerol present in the sample before hydrolysis. For this purpose 10 mg/dL (0.11 mmol / L) was subtracted from the result obtained.

**Apolipoprotein A1 (Apo-A1).** Apo-A1 was determined by the immunoturbidimetric method. The procedure was carried out following the manufacturer’s instruction (DiaSys Germany). Apo-A1 reacted with antibodies against Apo-A1, forming insoluble complexes. The degree of turbidity was measured at 580 nm.

**Apolipoprotein B (Apo-B).** Apo-B was determined by immunoturbidimetry. The procedure was carried out following the manufacturer’s instruction (DiaSys Germany). Apo-B reacted with antibodies against Apo-B, forming insoluble complexes. The degree of turbidity was measured at 340 nm.

**Equipment for biochemical measurements.** Measurements of TAS, TC, HDL-C, TG, Apo-A1 and Apo-B concentrations were performed on an auto-analyzer (Konelab 20i Thermo Fisher Scientific, USA). Concentrations of VC, VE, 4-HNE were determined using the microplate reader Multiskan GO (Thermo Fisher Scientific, USA). Concentrations of CD, MDA and TOS were measured with a UV-6300PC spectrophotometer (VWR International, China).

### Statistical analyses

**Fig 1:**
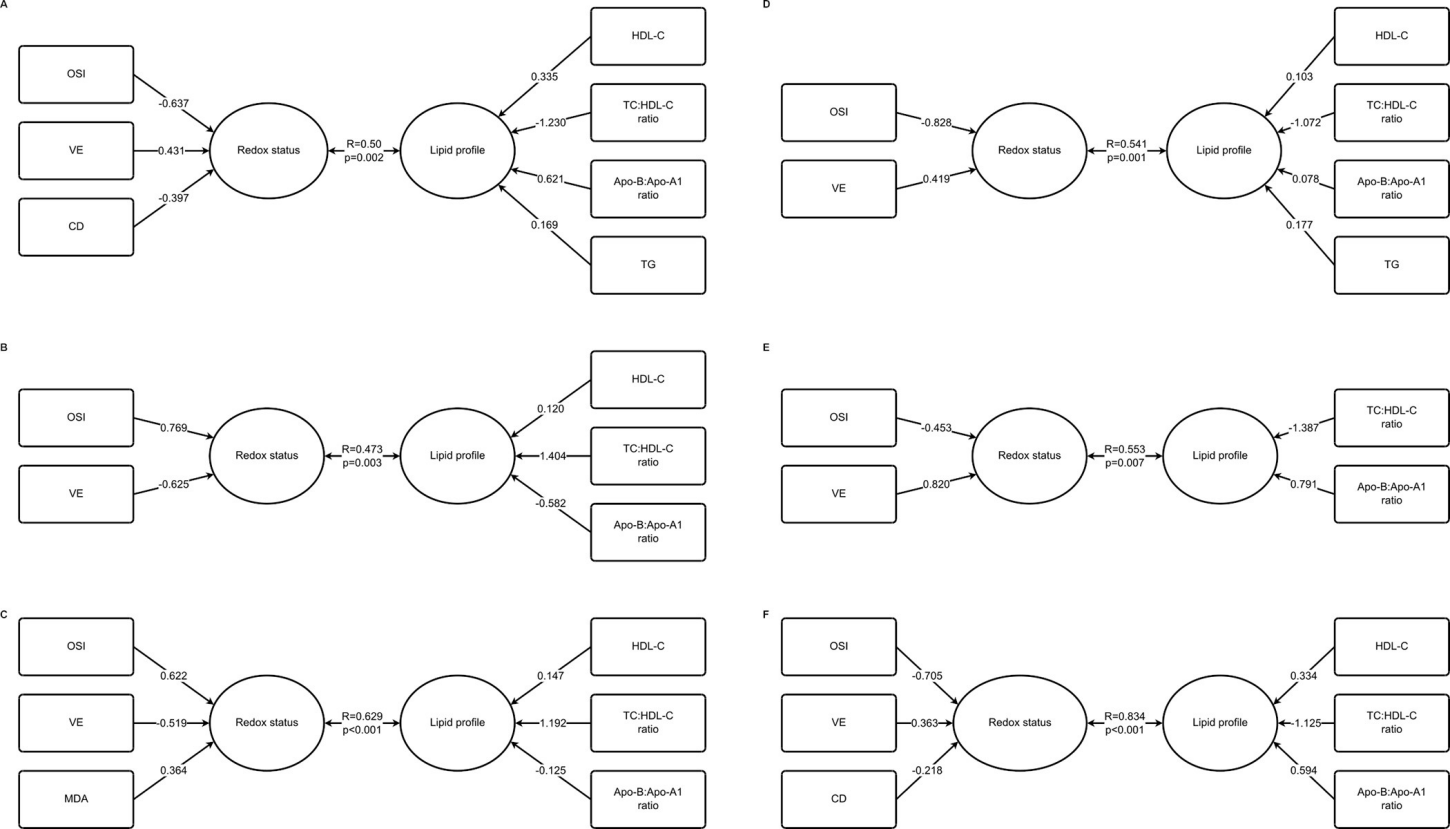
Canonical analyses between parameters related to redox status and serum lipid metabolism in all LC patients (A), male (B), general overweight/obese (C), abdominal obesity (D), non-smoking (E) and in LC patients with hypertriglyceridemia (F).

The data were analyzed using Statistica 12, PL (StatSoft). The chi-square test was used for intergroup comparisons of smoking status, alcohol consumption, chemical agent exposure, education level and place of residence. To evaluate the differences in age, concentrations of parameters related to lipid metabolism and redox status, as well as in dietary intakes and in results of anthropometric parameters between lung cancer and the control sex-related groups, Student’s t test (parametric data) or the Mann-Whitney U-test (non-parametric data) were performed. Shapiro–Wilk test was used for testing the normality of data in every group or sub-group included in the analyses. All data concerning biochemical variables, and anthropometric parameters, as well as data on dietary intake and age were tested. Normally distributed data were those with p>0.05 in the Shapiro-Wilk test. Data are presented as median (Q1-Q3) in connection with the high proportion of non-normal distribution. Additionally, due to partially non-normally distributed data, Box-Cox transformation was used and missing data were replaced by the respective group average before correlation analyses (Pearson and canonical correlations). The highest percentage of replaced missing data is 5.7% for vitamin E and 4-HNE, followed by 5.2% for OSI, 4.6% for VC, TAS, 2.3% for MDA and CD, 1.7% for TOS and 1.1% for lipid parameters. Correlation analyses were performed for all lung cancer patients as well as in sub-groups separated based on sex, smoking status (yes/ no/ previous smoking), general overweight or obesity assessed with BMI (≥ 25.0 kg/m^2^), abdominal obesity assessed with WHR > 0.85 for women and > 0.90 for men, and hypertriglyceridemia (TG>150 mg/dl). To discover general relationships between two sets of serum variables related to lipid metabolism and redox status, canonical analyses were performed. In this study, the use of canonical analysis to test data was mainly aimed at discerning the interrelations between the linear combinations of the first data set (lipid metabolism parameters) and the second data set (redox status parameters). The essence of the technique is to derive a linear combination for each of multiple data sets in such a way that the correlation between these two linear combinations is maximized. First, we performed simple correlation analyses (Pearson correlations) to find the most correlated variables from both multivariate sets of variables (vectors). Then we kept building different models of canonical analysis until we found the strongest linear correlation between the two collections of parameters of lipid metabolism and redox status. We chose only one model for each subgroup of patients, with the highest canonical correlation coefficient, to group the relationships into a lesser number of statistics while preserving the main facets of the relationships (dimension reduction technique). To show how great a respective variable's unique contribution to the sum was, we used canonical weights (presented in Fig 1).

For all statistical procedures, the significance level was considered to be < 0.05.

## Results

Baseline characteristics of lung cancer patients and control subjects based on clinical and nutrition data and anthropometric parameters are included in Table 1. Patients were heterogeneous in terms of histological type of lung cancer and clinical stage of disease, although there were no differences between lung cancer women and men. The majority of them, regardless of sex, were diagnosed with non-small cell lung cancer (NSCLC), about one-third of women had stage I disease, 20%—stage II and less than 10% of women had stage IV disease, while in men similar percentages had stage I or IV disease–ca. a quarter of them, while every fifth was at stage II. Smoking status did not differ between lung cancer and control sex-related groups, while less percentage of lung cancer men declared regularly consumption of alcohol. Moreover significantly more lung cancer women were worse educated and higher percentage of lung cancer patients than control subjects lived in small city, regardless of sex. Lung cancer patients were additionally characterized in terms of chronic comorbidities and used medicines. The most frequently co-occurring disease was hypertension, followed by type 2 diabetes mellitus (T2DM) and dyslipidemia. The most often chronically used agents were as follows: antihypertensive and antidiabetic ones.

Lung cancer patients were comparable to control subjects in terms of anthropometric parameters, except for a significantly lower weight of lung cancer men and a significantly higher WHR of lung cancer women as compared to the respective sub-groups of control subjects.

Data on nutrient intakes revealed significantly lower diet energy in control subjects compared to lung cancer patients, regardless of sex. Additionally, lung cancer men had significantly higher amounts of total carbohydrates, total fats and monounsaturated fatty acids (MUFAs) in their diets compared to control subjects, while lung cancer women consumed significantly higher amounts of saturated fatty acids (SFAs) in comparison with control women. We did not demonstrate differences in total protein intakes, cholesterol, polyunsaturated fatty acids (PUFAs) or dietary vitamin C between lung cancer and control subjects, regardless of sex. However, dietary vitamin E and retinol intakes were significantly higher in lung cancer than control men and women, respectively, while B-carotene intakes were significantly higher in control than lung cancer women.

Results concerning serum parameters of lipid metabolism and systemic redox status of lung cancer patients and control subjects are presented in Table 2.

**Table 2:** Serum parameters of lipid metabolism and systemic redox status of sex-related groups of lung cancer patients (n = 92) and control subjects (n = 82) [median (Q1-Q3)].

Biochemical parameters	Women				Men			
	N	Lung cancer (n = 43)	n	Control (n = 42)	N	Lung cancer (n = 49)	n	Control (n = 40)
	**Serum parameters of lipid metabolism**							
**T-C [mg/dl]** ^**W**^	41	199.0 (165.8–230.2)**	42	232.5 (196.0–256.2)	49	190.0 (155.7–225.7)	40	202.8 (184.2–238.7)
**LDL-C [mg/dl]**	41	123.5 (102.6–148.6)*	42	147.6 (123.0–182.4)	49	130.5 (98.2–155.4)	40	135.1 (110.9–174,8)
**HDL-C [mg/dl]** ^**W**^	41	42.6 (34.6–50.1)***	42	52.2 (46.1–60.5)	49	33.4 (25.0–41.4)***	40	44.1 (36.5–50.7)
**T-C : HDL-C ratio**	41	4.39 (3.63–5.56)	42	4.3 (3.54–5.04)	49	5.85 (4.49–6.87)*	40	5.03 (3.99–5.96)
**Non-HDL-C [mg/dl]**	41	147.3 (122.5–177.5)	42	171.6 (140.6–208.0)	49	155.9 (123.6–187.7)	40	160.2 (137.4–199.8)
**TG [mg/dl]**	41	130.9 (103.0–158.4)*	42	102.3 (84.2–139.6)	49	121.7 (90.5–157.0)	40	113.3 (75.2–156.1)
**Apo-A1 [mg/dl]** ^**W,M**^	41	155.6 (126.4–177.8)**	42	176.8 (159.6–198.0)	49	133.0 (108.9–156.4)***	40	165.7 (146.6–188.0)
**Apo-B [mg/dl]** ^**W,M**^	41	75.6 (59.5–91.2)**	42	90.3 (79.2–108.2)	49	81.5 (68.2–95.4)*	40	89.3 (74.2–112.2)
**Apo-B: Apo-A1 ratio**	41	0.49 (0.38–0.59)	42	0.52 (0.42–0.65)	49	0.59 (0.50–0.74)	40	0.56 (0.42–0.71)
	**Systemic redox status**							
**TAS [mmol Trolox equiv./L]** ^**W,M**^	38	1.66 (1.43–1.76)	42	1.66 (1.55–1.72)	46	1.61 (1.41–1.78)***	40	1.80 (1.67–1.93)
**TOS [mmol H**_**2**_**O**_**2**_ **equiv./L]**	41	3.07 (1.46–5.18)	42	2.22 (1.75–3.35)	48	3.70 (2.33–23.9)	40	3.08 (1.85–5.19)
**OSI [arbitrary unit]**	38	1.86 (0.91–2.88)	42	1.40 (1.04–2.25)	45	2.12 (1.50–14.9)*	40	1.82 (1.04–2.69)
**VC [μmol/L]**	40	1.37 (1.0–2.43)*	42	2.03 (1.27–4.80)	44	1.28 (1.10–1.48)	40	1.41 (1.19–1.84)
**VE μg/mL**	40	13.0 (8.97–18.6)***	42	27.2 (17.1–36.8)	44	12.1 (8.73–16.3)***	38	25.4 (19.2–38.3)
**MDA [μmol/L]**	41	1.51 (1.15–2.11)	41	1.36 (1.19–1.66)	49	1,81 (1.37–2.34)	39	1.90 (1.68–2.54)
**4-HNE [ng/mL]**	40	31.87 (17.66–44.53)***	42	14.82 (12.23–27.90)	42	19.97 (14.62–37.21)*	40	13.76 (12.55–23.82)
**CD [μmol/L]**	42	56.1 (43.1–74.1)***	42	34.4 (24.9–47.2)	47	60.0 (53.3–91.4)***	39	45.4 (30.7–61.8)

T-C–total cholesterol; LDL-C–low-density lipoprotein cholesterol; HDL-C–high-density lipoprotein cholesterol; TG–triglycerides; Apo-A1 –apolipoprotein A1; Apo-B–apolipoprotein B; TAS–total antioxidant status; TOS–total oxidant status; OSI–oxidative stress index; VC–vitamin C; VE–vitamin E; MDA–malonyldialdehyde; 4-HNE– 4-hydroxynonenal; CD–conjugated dienes; significant differences in biochemical parameters between sex-related groups are bolded

*p<0.05

** p<0.01

***p<0.001, differences were evaluated with t-Student or U-Mann-Whitney test depending on data distribution.

^W,M^–women, men—sex-related groups for whom parameters evaluated with t-Student test (normally distributed) are marked in the first column.

We observed a significant decrease in parameters related to serum cholesterol in lung cancer patients when compared with control subjects. Lung cancer women had significantly lower TC and LDL-C, while HDL-C and Apo-A1 and Apo-B were decreased in lung cancer patients, regardless of sex. Additionally, TC:HDL-C ratio was significantly increased in lung cancer men when compared to control men, while TG were higher in lung cancer women than in control women. Non-HDL-C as well as Apo-B:Apo-A1 ratios were comparable between sex-related groups of lung cancer and control subjects.

Data on parameters of systemic redox status revealed important disorders in antioxidant/prooxidant balance in lung cancer patients when compared to control subjects. TAS was significantly lower, while the index of oxidative stress (OSI) was increased in lung cancer subjects when compared to control ones, but only in male sub-groups. Additionally, we observed significant depletion of serum antioxidant vitamin concentrations. VE and VC were twice as low in lung cancer patients when compared to control subjects, but differences in VC affected only women. Parameters of lipid peroxidation, CD and 4-HNE, were significantly increased in lung cancer subjects as compared to control ones, except for serum concentrations of MDA, which were comparable between groups.

Correlations between parameters of systemic redox status and those related to lipid metabolism, observed in all lung cancer patients (All LC), as well as in the sub-groups of patients separated based on sex (lung men—LM and lung women—LW), smoking status (former smoking–FS and non-smoking–NS), hypertriglyceridemia (>TG), general overweight, obesity (>BMI) and abdominal obesity (>WHR), are presented in Table 3. We did not observe any positive relationships between antioxidant parameters like VC, VE, TAS and those characterizing lipid metabolism. All significant relationships with lipid parameters for antioxidant variables were negative and, except for the one between TAS and Apo-B:Apo-A1 ratio, were demonstrated mainly for VE with T-C:HDL-C ratio, non-HDL-C, TG and Apo-B. Moreover, the correlation coefficients (R) obtained for those relationships were higher in particular subgroups of patients: those who were general overweight or obese, with abdominal obesity, and those with hypertriglyceridemia. The highest number of correlations of parameters of lipid metabolism were obtained from the parameters of oxidative stress (TOS and OSI) and fewer were found for the parameters of lipid peroxidation (4-HNE and CD). A multitude of correlations were observed in all lung cancer patients as well as different subgroups of them for HDL-C (negative) as well as for T-C:HDL-C ratio (positive) with TOS, OSI and to a lesser extent with 4-HNE and CD. The relationships concerning TOS and OSI were stronger in former or non-smoking patients, as well as in subgroups of patients with general overweight/obesity and/or with abdominal obesity, while those for 4-HNE and CD–in non-smoking patients. Almost all the above- mentioned correlations were strongest in the subgroup of patients with hypertriglyceridemia (R> 0.50). Many correlations were also observed for Apo-A1 and Apo-B:ApoA1 ratio. Apo-A1 negatively correlated with TOS, OSI and CD–mainly in the subgroup of patients with hypertriglyceridemia and, surprisingly, positively with 4-HNE–mainly in former smoking patients, while Apo-B:Apo-A1 ratio correlated positively with parameters of oxidative stress (TOS and OSI) and negatively with 4-HNE, but only in formerly smoking patients. Correlations for Apo-B:Apo:A ratio with parameters of oxidative stress were strongest in patients with hypertriglyceridemia followed by patients with abdominal obesity and general overweight/obesity. Interestingly, TC and LDL-C correlated only with MDA concentration, and those relationships were rather weak: R<0.3 for general overweight/obese patients and R<0.4 only for a subgroup of women. We did not observe any correlations for subgroups of current smoking lung cancer patients.

**Table 3:** Statistically significant correlations between serum parameters of lipid metabolism and parameters related to systemic redox status in all lung cancer patients (all LC) and the following subgroups: lung cancer women (LW), lung cancer men (LM), patients with general overweight/obesity (>BMI), abdominal obesity (>WHR), hypertriglyceridemia (>TG), and those who were non-smokers (NS) and former smokers (FS).

	T-C	LDL-C	HDL-C	TC:HDL-C ratio	Non-HDL-C	TG	Apo-A1	Apo-B	Apo-B:Apo-A1 ratio
**Correlations with parameters related to systemic antioxidant status**									
**TAS**									-0.22*(all LC)
** **									-0.32*(LW)
**VE**				-029*(all LC)	-0.28(>BMI)	-0.30*(LM)		-0.26*(>WHR)	
** **				-0.30*(LW)	-0.26*(>WHR)	-0.30*(>BMI)			
** **				-0.41**(>BMI)		-0.24*(>WHR)			
** **				-0.34**(>WHR)					
** **				-0.39*(>TG)					
**Correlations with parameters related to systemic oxidant status and lipid peroxidation**									
**TOS**			-0.34* (all LC)	0.35*(all LC)			-0.52**(>TG)	0.34*(>BMI)	0.39**(>BMI)
** **			-0.34*(LW)	0.35*(LM)				0.27*(>WHR)	0.41**(>WHR)
** **			-0.39*(NS)	0.35*(FS)					0.54**(>TG)
** **			-0.39**(>BMI)	0.36*(NS)					
** **			-0.42***(>WHR)	0.49***(>BMI)					
** **			-0.69***(>TG)	0.46***(>WHR)					
** **				0.75***(>TG)					
**OSI**			-0.34* (all LC)	0.36***(all LC)	0.28*(>BMI)	0.27*(>BMI)	-0.26*(>WHR)	0.29*(>BMI)	0.22*(all LC)
** **			-0.32*LM)	0.36*(FS)			-0.50**(>TG)		0.40**(>BMI)
** **			-0.39* (FS)	0.37**(LM)					0.41***(>WHR)
** **			-0.39**(>BMI)	0.42*(NS)					0.41***(>WHR)
** **			-0.41***(>WHR)	0.47***(>WHR)					0.54**(>TG)
** **			-0.69***(>TG)	0.50***(>BMI)					
** **				0.76***(>TG)					
**MDA**	0.28*(>BMI)	0.34*(>BMI)		0.27*(>BMI)	0.31*(>BMI)				
** **	0.36*(LW)	0.37*(LW)		0.35*(LW)	0.38*(LW)				
**4-HNE**			0.43*(FS)	-0.37*(FS)			0.25*(all LC)		-0.42*(FS)
** **			-0.40*(NS)	0.40*(NS)			0.26*(>WHR)		
** **							0.44**(FS)		
**CD**			-0.24*(>WHR)	0.21* (all LC)			-0.22*(all LC)		
** **			-0.41*(NS)	0.37*(NS)			-0.38*(NS)		
** **			-0.58**(>TG)				-0.59**(>TG)		

*<0.05

**<0.01

***<0.001 –correlation analyses were performed with a Pearson correlation test after making data normally distributed using Box-Cox transformation and replacing missing data with respective group averages

T-C–total cholesterol; LDL-C–low-density lipoprotein cholesterol; HDL-C–high-density lipoprotein cholesterol; TG–triglycerides; Apo-A1 –apolipoprotein A1; Apo-B–apolipoprotein B; TAS–total antioxidant status; VE–vitamin E; TOS–total oxidant status; OSI–oxidative stress index; MDA–malonyldialdehyde; 4-HNE– 4-hydroxynonenal; CD–conjugated dienes; all LC–all lung cancer patients; LW–lung cancer women; LM–lung cancer men; >BMI–lung cancer patients with overweight or obesity assessed with BMI; >WHR–lung cancer patients with abdominal obesity assessed with WHR; >TG–lung cancer patients with TG>150 mg/dl; FS–formerly smoking lung cancer patients; NS–non- smoking lung cancer patients

In order to find relationships between two sets of variables, i.e. biomarkers of redox state and those related to lipid metabolism, and based on the individual associations found with Pearson correlation analyses, presented in Table 3, we built models of canonical analyses which were stratified by sex, general overweight/obesity, abdominal obesity and hypertriglyceridemia as well as smoking status, and these are presented in Fig 1. In general, regardless of the type of model, the most important parameters of redox status and systemic lipid metabolism determining relationships between these two phenomena were, respectively: OSI, VE and HDL-C, TC:HDL-C ratio as well as Apo-B:Apo-A1 ratio. Additionally, in the group of all lung cancer patients, as well as in patients with hypertriglyceridemia, CD significantly contributed to the models of relationships found. In patients with general overweight/obesity, MDA additionally influenced the observed relationship, while in all lung cancer patients and lung cancer patients with abdominal obesity, levels of TG affected the model of observed dependencies. Among all the models built, the highest canonical correlation coefficient was observed for that found in patients with hypertriglyceridemia (R = 0.834), followed by that for general overweight/obese patients (R = 0.629), non-smoking ones (R = 0.553), abdominal obese (R = 0.541), all patients (R = 0.490) and for lung cancer men (R = 0.473). Regardless of the model, OSI presented the highest absolute value of canonical weight in the set of redox variables, except for the model presented for non-smoking lung cancer patients, where VE had the highest absolute value of canonical weight. In the set of variables related to lipid metabolism, TC:HDL-C ratio presented the highest absolute value of canonical weight, followed by Apo-B:Apo-A1 ratio in the following models built for the group of all patients, male lung cancer patients, non-smoking ones and those with hypertriglyceridemia. In the model presented for general overweight/obese lung cancer patients Apo-B:ApoA1 ratio and HDL-C had a similar absolute value of canonical weight, while for the group of patients with abdominal obesity Apo-B:Apo-A1 ratio had the smallest absolute value of canonical weight, and higher values were observed for TG and HDL-C.

## Discussion

During cancer development and progression, tumor cells activate or up-regulate many different metabolic pathways that change the metabolism of carbohydrates, proteins and lipids to ensure three basic tumor features: generating ATP, undertaking biosynthesis, which in turn supports tumor progression, and maintaining redox balance to allow tumor cells to stay alive [19]. Over the past decades many studies have been performed to determine alterations during cancer development in metabolic pathways at cellular as well as systemic levels. The majority of them have been devoted to carbohydrate metabolism and produced the main ideas concerning glucose turnover–the Warburg effect, the Cori cycle, and the tumor-stroma model [19]. In vitro studies indicated that tumor cells regulate redox balance and counteract oxidative stress triggered by changes in glucose metabolism [20]. However, our previous studies showed that changes in carbohydrate metabolism expressed as alterations in systemic biochemical parameters might also be related to systemic redox alterations [3]. To our best knowledge, at the systemic level such relationships were demonstrated for the first time in oncological disorders. Therefore based on our previous studies, we hypothesized that alterations in lipid metabolism reflected in changes in systemic lipid parameters could also be linked with systemic disorders in antioxidant/pro-oxidant homeostasis in lung cancer. Such associations have not been investigated in lung cancer before, or in any other cancerous disease, but they have been shown in many other non-cancerous diseases, mainly in atherogenic and diabetic conditions as well as chronic kidney disease [21–23].

Alterations in lipid metabolism during cancerogenesis have been extensively studied over the last decades. Experimental studies have proven a predisposition to lipid alterations during cancerogenesis and/or cancer proliferation [24]. In the cellular tumor milieu, lipid synthesis is reactivated *de novo*. Moreover, in some cancers, including lung cancer, multifunctional enzyme fatty-acid synthases are overexpressed, indicating poor prognosis due to significantly increased cancer aggressiveness [24,25]. On the other hand, as demonstrated in many studies, malignant transformation and cancer cell proliferation are intimately related to alterations in systemic lipid parameters, with contradictory results concerning the observed associations [7,26–28]. Some of them are well-known risk factors of cancer prevalence while others–a consequence of disease development and/or progression. For example, Siemianowicz et al. [28] showed that lung cancer patients are characterized by significantly decreased TC and TG levels. In the last meta-analysis performed by Lin et al. [7] it was confirmed that low TC and high TG concentrations created a predisposition to higher lung cancer incidence, while Kucharska-Newton et al. [9] indicated that lung cancer patients had lower HDL-C, which is significantly but weakly linked with lung cancer incidence. Additionally, in their latest study, Zhou et al. [10] proved that low serum LDL-C concentration was a favorable prognostic factor for overall survival in patients with small-cell lung cancer. In our study we found that HDL-C was decreased and TG increased in lung cancer patients as compared to control subjects, regardless of sex, while TC and LDL-C were lower but only in lung cancer women in comparison with the control women. Our results are in agreement with the studies presented above, but only Kucharska-Newton et al. [9] stratified their results according to sex. Additionally, we found that Apo-A1 and Apo-B were decreased in lung cancer patients compared with control subjects, regardless of sex. There are a handful of studies concerning associations between Apo-A1, Apo-B and lung cancer incidence. In an epidemiological study, Borgquist et al. [29] indicated that serum Apo-A1 was inversely associated with lung cancer risk, regardless of sex, while Apo-B positively correlated with lung cancer incidence in both sexes. The discrepancies in the results of Apo-B obtained in our study and the above-mentioned one [29] may be the result of concentrations of LDL-C. There are divergent data on associations between Apo-B and LDL-C, although it is generally recognized that the concentration of Apo-B is related to LDL-C level [30]. However, in our study, serum Apo-B concentration was decreased in lung cancer patients, regardless of sex, while LDL-C concentration was reduced only in the sub-group of women. No differences were found in Apo-B:Apo-A1 ratio between lung cancer and control sex-related sub-groups, although in the Borgquist et al. study [29] ApoB:Apo-A1 ratio conferred an increased lung cancer incidence. Multifactorial alterations in lipid metabolism observed in lung cancer patients may be a risk factor as well as a consequence of cancer development. In general they result from e.g. altered food intake, which increases lung cancer risk, reduced cancer-related lipogenesis in the liver, and enhanced lipid mobilization from adipose tissue, which are direct consequences of cachexia and/or other cancer-related metabolic abnormalities [31]. Based on nutrient intake assessment, we have observed the following differences in fatty nutrient contents between lung cancer and control subject diets: higher content of SFAs in lung cancer women’s diets and higher total fat as well as MUFA content in the diets of men with lung cancer. In the latest epidemiologic study performed as a pooled analysis of several prospective cohort studies, it was revealed that high intakes of total and SFAs were associated with an increased risk of lung cancer, but no associations were observed for MUFAs [32]. The authors additionally detected synergistic interaction between smoking and total fat intakes in relation to lung cancer risk. The proposed possible mechanism of this interaction is based on the enhanced activity of nicotine-derived nitrosamine ketone (NNK), which promotes lung cancerogenesis by a high content of fat in the diet [32]. Indicated differences in nutrient intakes in this study could have influenced the observed systemic lipid alterations. However, the other factors mentioned above should also be considered, as there is a great probability, in particular that metabolic disorders and cachexia often occur in the course of lung cancer and may also affect lipid metabolism, in turn, as a consequence of cancer development [33]

Our study demonstrated alterations in systemic redox status, both in particular parameters and in indicators of total oxidant or antioxidant status. Additionally, the observed disturbances were sex-related. In general, we found that disturbances in redox status were more disclosed in lung cancer men than women. Although VE was decreased in all lung cancer patients, regardless of sex, and therefore lipid peroxidation was intensified in lung cancer men as well as women, only in men did we observe depletion of the antioxidant capacity of serum, and therefore a significant increase in systemic oxidative stress. Disturbances in redox status in lung cancer are well-known phenomena; however studies concerning systemic parameters and particularly those reflecting the sum and interactions between individual parameters and related to gender, are scarce [3,6,34]. The impact of gender has also been demonstrated in other studies regarding elderly people, both healthy and with chronic diseases, but the results are contradictory [35,36]

Lipid peroxidation is a major concern due to its ability to generate a large variety of breakdown products including alkanes, aldehydes, ketones, furans and others. Aldehydes receive a lot of attention due to their high reactivity. Among them, MDA is quantitatively a major product of lipid peroxidation with a high potential for mutagenicity mainly due to genotoxicity through forming guanosine, cytidine and adenosine adducts. Another product of lipid peroxidation, 4-HNE, is strongly related to concentrations of n-6 polyunsaturated fatty acids. Although 4-HNE appears in smaller concentrations than MDA, its importance is considerably higher due to its implication in pathological conditions such as cancers [37]. However, regardless of peroxidation product type, MDA as well as 4-HNE may generate point mutations in tumor suppressor genes, and therefore may significantly increase the risk of inflammatory-related cancers such as lung cancer [38]

In previous studies lipid peroxidation in lung cancer patients was assessed based mainly on MDA concentration. Indeed, many studies have confirmed that systemic MDA concentrations are increased in lung cancer patients [39,40].

In contrast to the presented results of other authors, we did not find significant differences in serum MDA concentration between lung cancer and control subjects. On the other hand, another parameter of lipid peroxidation, 4-HNE, was significantly increased in lung cancer subjects, regardless of sex. Additionally, we observed that differences in 4-HNE concentrations between cancerous and control subjects were more apparent in women than men, which additionally indicates that lipid peroxidation might be more intense in female than male cancer patients. There are ambiguous data on dependencies between 4-HNE and tumor development. In gastric cancer [41] serum 4-HNE concentration was significantly decreased compared to healthy subjects, while a significant increase in 4-HNE concentration was observed in lung cancer, but at the tissue level [42]. 4-HNE is widely accepted as an inducer and/or mediator of oxidative stress. It may strongly affect redox status in the cellular milieu by exhausting cellular sulfhydryl compounds such as glutathione. On the other hand, at concentrations slightly above endogenous levels, this molecule may play an important role in signaling pathways, due to transcriptional and gene expression changes [43]. Despite this either cytoprotective or cytotoxic role, 4-HNE is recognized to initiate and cause the progression of cancers, mainly as a mitochondrial–protein, lipid, DNA or other molecules containing nucleophilic thiol or amino groups–adduct [44,45].

The elevated concentrations of CD in lung cancer patients, regardless of sex, observed in our study did not confirm the results of Crohns et al. [46] who indicated no changes in systemic conjugated dienes between lung cancer and control subjects. However, several other studies concerning different types of cancers such as breast [40], cervical [47] and ovarian [48] were in agreement with our results. Moreover, research on changes in lipid peroxidation during tumorigenesis in pulmonary tissue also showed a significant increase in CD in cancer tissue homogenates [49]. In contrast, in the Ma et al. study [41] CD concentrations in sera of gastric cancer patients were significantly decreased when compared to healthy controls.

The link between systemic lipid metabolism and redox status in cancer diseases is still poorly understood, although some studies have indicated the potential contribution of lipid disorders to oxidative stress. To our best knowledge this is the first study emphasizing the relevance of relationships between the above-mentioned phenomena in lung cancer at the systemic level. In this study, observed alterations in serum lipid parameters, as well as redox balance were interdependent, as was shown based on simple Pearson correlations as well as a multidimensional statistical method—canonical analyses.

The majority of associations with antioxidant variables in this study were found mainly for VE. Vitamin E is able to counteract lipid peroxyl radicals, thus terminating the peroxidation chain reaction, and thereby reducing oxidative damage. It is the major lipophilic, radical-scavenging antioxidant *in vivo*, protecting against oxidative stress mediated by active oxygen as well as nitrogen species [50]. A decrease in VE concentration was observed in this and other studies concerning lung cancer patients. Gackowski et al. [51] also demonstrated that serum alpha-tocopherol was lowered in lung cancer patients compared to smoking as well as non-smoking control subjects. Moreover, in other types of cancer, e.g. in stomach, colon, rectal and breast cancers, depletion of serum alpha tocopherol was also observed [52]. In this study, the observed decreased of serum VE did not result from vitamin E intakes which were comparable between lung cancer and control women, while in the group of lung cancer men, dietary intakes of this vitamin were even higher in comparison to control men. Therefore, the significant drop in serum concentration was probably related to oxidative stress mediated by the development of cancer and/or alterations in metabolism. Another potential mechanism explaining the decreased VE concentration may involve alterations in alpha-tocopherol distribution. Circulation of alpha-tocopherol in the blood is principally within the LDL-C fraction–as the serum lipids increase, alpha-tocopherol seems to partition out of the cellular membrane compartment into circulating lipoproteins [52]. Therefore in patients with low LDL-C concentration, e.g. in cancer patients, alpha-tocopherol might be decreased. However, we found no positive associations between VE and LDL-C, and further, negative correlations were observed for non-HDL-C and TC:HDL-C ratios.

A low concentration of VE creates a predisposition to higher concentration of parameters related to dyslipidemia. Therefore in our study we showed that VE negatively correlates with TC:HDL-C ratio, as well as non-HDL-C and TG. Similar results were obtained in an experimental study performed by da Costa et al. [53] on spontaneously hypertensive rats as well as in a clinical study by Amini et al. [54] with patients suffering from dyslipidemic disorders. In the study by da Costa et al. [53], the authors revealed that treatment with alpha-tocopherol led to a decrease in LDL-C and an increase in HDL-C, while TG and T-C were unchanged. On the other hand, Amini et al. [54] in a study with dyslipidemic patients found that serum VE concentrations were negatively correlated with TC, LDL-C, and TG, as well as positively with HDL-C.

In this study, we found that oxidative stress in lung cancer patients was strongly and significantly associated with lipid disturbances. We observed that oxidative stress index and total oxidant status were increased mainly along with the increase in TC:HDL-C ratio but also with the decrease in HDL-C concentrations. Additionally, Apo-B:Apo-A1 ratio intensified oxidative stress. The results concerning associations between oxidative stress and lipid alterations presented in our study are in agreement with those obtained by other authors in different populations. In the Yang et al. [22] study, MDA–a marker of oxidative stress–was significantly increased in hyperlipidemic patients when compared to normolipidemic ones. Additionally, MDA significantly positively correlated with atherogenic index. In our study the parameters of lipid peroxidation MDA and CD were also positively associated with those reflecting dyslipidemia, i.e. TC, LDL-C, non-HDL-C, as well as TC:HDL-C ratio, and negatively with HDL-C and Apo-A1. The mechanism of these associations is based on oxidation of PUFAs within LDL-C and then the breakdown of oxidated fatty acids resulting in the formation of aldehydes and ketones such as MDA or 4-HNE [55].

The negative associations between HDL-C and oxidative stress revealed in this study may result mainly from the antioxidant properties of HDL-C. The antioxidant activity of HDL-C is mainly mediated by the content of the enzyme esterase–paraoxonase-1 (PON-1). The exact mechanism of the protective activity of this enzyme has not been well established but most likely results from the peroxidase-like activity of PON. Therefore during the hydrolyzation of preformed lipid peroxides, PON-1 may inhibit or at least delay the initiation of oxidation [12]. Moreover, under normal conditions, HDL-C counteracts the formation and progression of plaque via reverse cholesterol transport and the prevention or slowing down of the oxidation of LDL-C [56]. In our study we observed strong negative associations between HDL, APO-A1 and parameters related to oxidative stress: mainly TOS and OSI.

The relationships discovered in this study were strengthened by several conditions, such as obesity (general, abdominal), hypertriglyceridemia, and non-smoking status. Obesity, especially the abdominal type, increases oxidative stress due to several molecular mechanisms. An increased content of ROS observed in adipose tissue is the result of local increased expression of NADPH oxidase, and additionally, although fat cells present many different antioxidant enzymes, the expression of SOD, catalase and GPX, the majority of them, is suppressed. The high content of ROS presented in adipose tissue is then released into the blood stream and therefore influences systemic redox balance towards oxidative stress. Additionally, visceral fat accumulation is strongly associated with systemic oxidative stress, even more than general adiposity [57]. The mechanism of oxidative stress induction by high TG level results from, often observed in this condition, high free-fatty acids concentrations which leads to increased mitochondrial-dependent oxidative stress [58]. Indeed, Cardona et al. [59] found significant and strong correlation between increased TG level after high-fat meal and parameters of oxidative stress: lipid peroxidation products (MDA and 4-HNE) and protein carbonyls and total nitrites, while correlations with antioxidant enzymes were inversely.

## Limitations and strengths of the study

Some limitations of the study should be considered when interpreting the data. The number of lung cancer patients and control subjects was limited and moreover lung cancer men were significantly older than control men. These limitations, as well as co-morbidity occurrence, hence chronically used medicines, could have influenced the results. Genetic abnormalities (polymorphisms, mutations) may at least partially determine lung cancer risk; therefore it might be worth evaluating the possible interaction of the observed associations with variations in genes related to antioxidants and lipids. Further studies are warranted to explore the mechanisms of the observed associations in detail, also at the cellular level, paying great attention to the tumor cell milieu, and in order to identify new pharmacological perspectives for lung cancer treatment.

The presented study, however, forms part of a group of multifaceted projects indicating links between redox status, an important phenomenon affecting both the development of lung cancer and its treatment sensitivity (mainly chemotherapy and radiotherapy), and endogenous factors like alterations in metabolism (in this study particularly lipid metabolism). Due to the significant contribution of redox status disorders in the molecular pathways of lung cancer development, the presented results may be of great practical value in preventive strategies. Stratifying the results by several conditions, such as overweight/obesity, hypertriglyceridemia and smoking status, showed their strong influence. Moreover, we are the first to demonstrate that in non-smoking lung cancer patients, the observed relationships between redox status and lipid metabolism are even stronger than in current or former smokers. The multitude of parameters of lipid metabolism routinely used in clinical practice and presented in this study provides the opportunity for further, more in-depth, studies of the presented relationships.

## Conclusions

Based on the obtained results, we demonstrated that parameters related to lipid alterations are associated with oxidative stress. The largest contribution from lipid parameters was revealed for TC:HDL-C ratio, HDL-C and Apo-B:Apo-A1 ratio, while the largest contributions from redox status were revealed for OSI and VE. General overweight, obesity and additionally abdominal obesity, as well as high TG levels, intensify these relationships. The relationships we obtained provide a basis for further studies in the field of factors modifying the redox status of lung cancer patients. Understanding these factors and the mechanisms of their redox modulating activity may lead to preventive strategies other than smoking cessation. Additionally, our findings may provide valuable insights for clinical settings and for the personalized treatment of lung cancer.

## Supporting information

S1 Data Dataset of the study. (CSV)
